# The antitumor natural product tanshinone IIA inhibits protein kinase C and acts synergistically with 17-AAG

**DOI:** 10.1038/s41419-017-0247-5

**Published:** 2018-02-07

**Authors:** Chao Lv, Hua-Wu Zeng, Jin-Xin Wang, Xing Yuan, Chuang Zhang, Ting Fang, Pei-Ming Yang, Tong Wu, Yu-Dong Zhou, Dale G. Nagle, Wei-Dong Zhang

**Affiliations:** 1 Shanghai Institute of Pharmaceutical Industry, China State Institute of Pharmaceutical Industry, Shanghai, 201203 P.R. China; 2 School of Pharmacy, Second Military Medical University, Shanghai, 200433 P.R. China; 3 School of Pharmaceutical Sciences, Zhengzhou University, Henan, 450001 P.R. China; 4 School of Pharmacy, Fujian University of Traditional Chinese Medicine, Fujian, 350108 P.R. China; 5 Institute of Interdisciplinary Integrative Biomedical Research, Shanghai University of Traditional Chinese Medicine, Shanghai, 201203 China; 6 Department of Chemistry and Biochemistry, College of Liberal Arts, University of Mississippi, University, Mississippi, MS 38677-1848 USA; 7 Department of BioMolecular Sciences and Research Institute of Pharmaceutical Sciences, School of Pharmacy, University of Mississippi, University, Mississippi, MS 38677-1848 USA

## Abstract

Tanshinone IIA (Tan IIA), the primary bioactive compound derived from the traditional Chinese medicine (TCM) *Salvia miltiorrhiza* Bunge, has been reported to possess antitumor activity. However, its antitumor mechanisms are not fully understood. To resolve the potential antitumor mechanism(s) of Tan IIA, its gene expression profiles from our database was analyzed by connectivity map (CMAP) and the CMAP-based mechanistic predictions were confirmed/validated in further studies. Specifically, Tan IIA inhibited total protein kinase C (PKC) activity and selectively suppressed the expression of cytosolic and plasma membrane PKC isoforms *ζ* and *ε*. The Ras/MAPK pathway that is closely regulated by the PKC signaling is also inhibited by Tan IIA. While Tan IIA did not inhibit heat shock protein 90 (Hsp90), it synergistically enhanced the antitumor efficacy of the Hsp90 inhibitors 17-AAG and ganetespib in human breast cancer MCF-7 cells. In addition, Tan IIA significantly inhibited PI3K/Akt/mTOR signaling, and induced both cell cycle arrest and autophagy. Collectively, these studies provide new insights into the molecular mechanisms responsible for antitumor activity of Tan IIA.

## Introduction

**Fig. 1:**
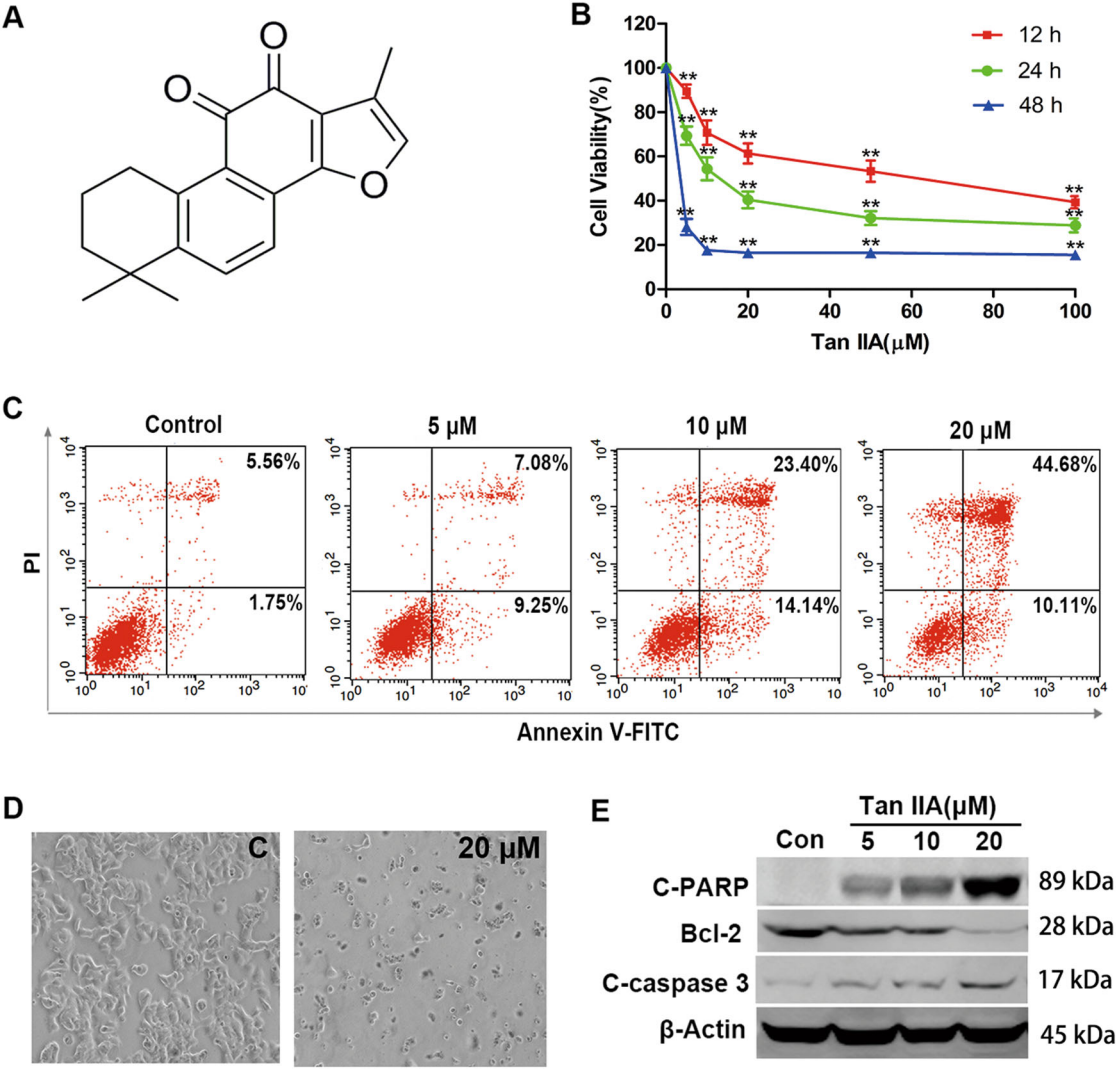
Tan IIA inhibited proliferation and induced apoptosis in MCF-7 cells. **a** Chemical structure of Tan IIA. **b** Viability of MCF-7 cells following Tan IIA treatment at the specified concentrations for 12, 24, and 48 h, respectively. Data shown are mean ± standard deviation of three independent experiments. **p < 0.01 when compared to the untreated control. **c** Quantification of annexin V-FITC/PI stained MCF-7 cells by flow cytometry to determine the extent of apoptosis following Tan IIA treatment. **d** Morphological changes of MCF-7 cells after treatment with Tan IIA (magnification, 100×). “C” represents control and 20 μM Tan IIA. **e** Effect of Tan IIA on the expression of apoptosis-related proteins (Bcl-2, cleaved PARP, and cleaved c-caspase 3) in MCF-7 cells, detected by western blot. The loading control is β-actin

Because of their ample chemical diversity and plethora of novel molecular targets, natural products are poised to remain a major source of new anticancer agents^1–3^. Tanshinone IIA (Tan IIA, Fig. 1a) is the major active compound from the root extracts of *Salvia miltiorrhiza* Bunge (Lamiaceae), which is prescribed for treating cardiovascular disease in the pharmacopoeia of the People’s Republic of China as the traditional Chinese medicine (TCM) “Danshen”. Tan IIA has been shown to exhibit anti-angiogenic, antioxidant, anti-inflammatory and antitumor activities. A number of studies support the potential of Tan IIA in treating liver^4^, cervical^5,6^, gastric^7^, colorectal^8^, prostate^9^, bladder^10^, and breast cancers^11^. Several omics-based approaches have been applied to predict the potential mechanism of action for Tan IIA. For example, functional proteomic analysis in combination with computational docking studies revealed that Tan IIA may interfere with microtubule assembly by binding to β-tubulin^6^. Integrated transcriptomics and proteomics studies suggested that Tan IIA inhibited gastric cancer cell growth by suppressing glucose metabolism^7^. While these studies provided a foundation of mechanistic Tan IIA data, the precise molecular antitumor mechanism(s) remains unclear.

In drug development, genome-wide gene expression profiling has frequently been employed to resolve the molecular mechanisms of drugs. New methods such as connectivity map (CMAP) database have emerged. To identify the mechanistic connections between small molecules, genes, and diseases, the CMAP database apply pattern-matching software to compare gene expression profiles and have made numerous advances in drug repurposing, target discovery and mechanism of action elucidation^12,13^. This CMAP database-based approach also has been successfully used to elucidate molecular mechanisms of TCMs (e.g., Si-Wu-Tang^14^) and TCM-derived compounds (e.g., berberine^15^). Thus, CMAP-based strategies can dramatically streamline the molecular mechanism prediction process for any given drug or other pharmacologically active substance.

The current version of CMAP database “build 02” (http://www.broadinstitute.org/cmap/) contains 6100 gene expression profiles derived from five different cell types treated with 1309 bioactive compounds^13^. However, most of compounds in the CMAP database are not derived from TCMs. Given the scarcity of TCM constituent-associated gene expression profiles, we established a database that contains the gene expression profiles for 102 TCM components, including Tan IIA^16^. These publicly available gene expression profiles provide an effective starting point to delineate the molecular mechanisms of TCM constituents. Herein, the gene expression profiles of Tan IIA-treated human breast cancer MCF-7 cells were analyzed using the CMAP method to identify potential molecular mechanisms of Tan IIA. Initial results suggested that Tan IIA may inhibit Hsp90, PKC, PI3K, and mTOR in cancer cells. The CMAP analysis-based predictions were further validated in vitro and in vivo. Our findings provide a new systematic and comprehensive view of the antitumor mechanisms of Tan IIA.

## Results

### Molecular mechanisms of Tan IIA predicted by a gene expression profile-based approach

**Fig. 2:**
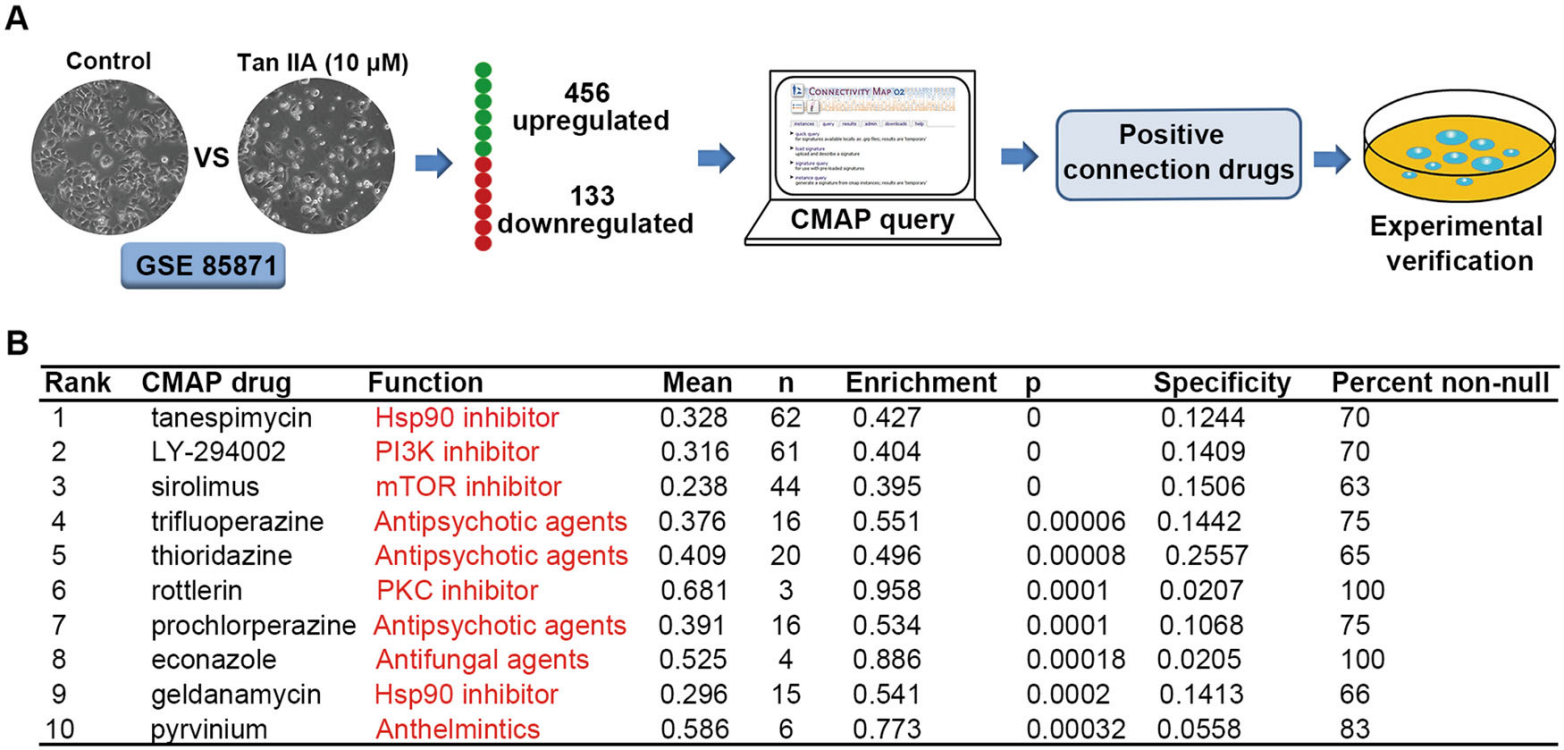
Gene expression profile-based prediction of Tan IIA’s molecular mechanisms. **a** Flow diagram of the CMAP analytical procedures. **b** Top ten Tan IIA-associated positive connection drugs/agents identified by the CMAP analysis

Previously, we established the gene expression profiles in response to 102 TCM components that serve as a general template for investigating molecular mechanisms^16^. Tan IIA (10 μM) treating MCF-7 cells produced the initial gene expression profiles in our data GSE85871 (https://www.ncbi.nlm.nih.gov/geo/) and Fold Change was used to filter the differential expression genes of Tan IIA. The query contained 589 genes (>2-fold change; 456 up-regulated and 133 down-regulated; Supplementary Data 1) that were submitted to the CMAP database for analysis (Fig. 2a). Top 10 correlated drugs with lower *p*-values and a positive enrichment score were calculated by CMAP database (Fig. 2b). Based on the known targets of these agents, it is likely that Tan IIA exerts its antitumor activity by inhibiting Hsp90, PKC, PI3K, and/or mTOR.

We also performed Kyoto Encyclopedia of Genes and Genomes (KEGG) pathway analysis of the Tan IIA using a multi-omics data analysis tool (OmicsBean)^17^ and the results are shown in supplementary data Fig. S1. Among the pathways identified, Ras signaling is activated by PKC or cross-talk with the PKC pathway^18,19^, the MAPK pathway acts downstream of the PKC cascade^20^, PI3K/Akt signaling and cell cycle progression are controlled by the PI3K/Akt/mTOR signaling pathway^21^. These findings were comparable to the results of CMAP analysis on potential molecular targets regulated by Tan IIA.

### Tan IIA exhibits anti-proliferative and antitumor activities in vitro and in vivo

In addition to serving as an in vitro model for microarray-based gene expression studies, MCF-7 cells were also employed to assess the antitumor activities of Tan IIA. In MCF-7 cells, Tan IIA suppressed cell proliferation/viability in a concentration- and time-dependent manner (Fig. 1b). Further, a concentration-dependent induction of apoptosis was observed in the presence of Tan IIA (Fig. 1c). Morphological examination revealed that Tan IIA reduced both the number and size of MCF-7 cells at the inhibitory concentration (Fig. 1d). To determine the extent of apoptosis, the levels of Bcl-2, caspase 3, and PARP proteins were monitored by western blot. After 24 h exposure to Tan IIA at the specified concentrations, a concentration-dependent decrease in the anti-apoptotic Bcl-2 protein was observed in MCF-7 cells, accompanied by an increase in the levels of cleaved caspase 3 and PARP proteins (Fig. 1e).

**Fig. 3:**
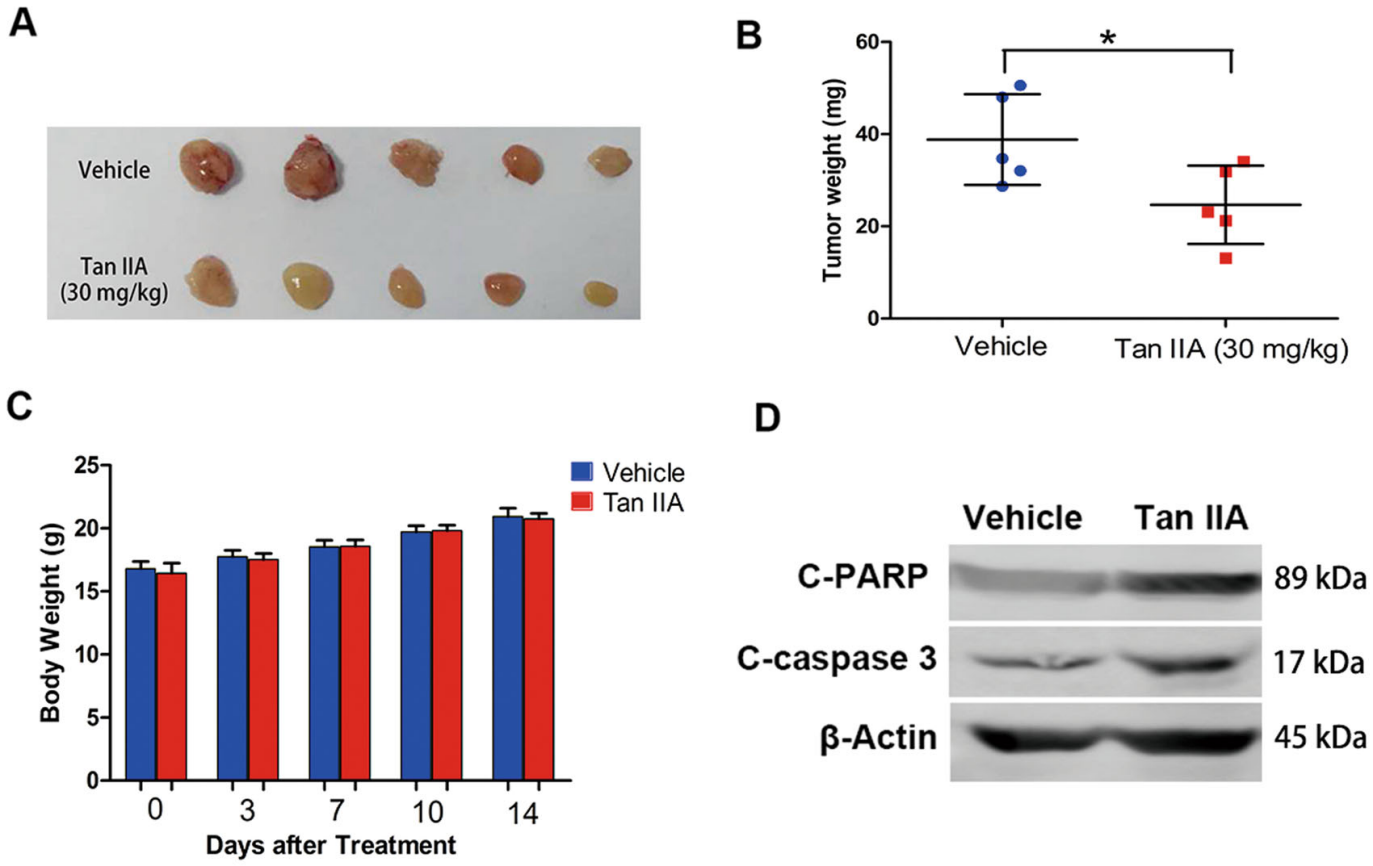
Tan IIA inhibited breast tumor xenograft growth in vivo. **a** Images of tumors excised from the control (vehicle) and Tan IIA-treated groups. **b** Tumor weight. Data shown are mean ± S.D. (five animals per group). *p < 0.05. **c** Body weight during treatment (day 0 represents the day that Tan IIA was administered). Data presentation and statistical analysis are the same as described in (**b**). **d** Total proteins were extracted from excised tumors and the levels of cleaved PARP and cleaved caspase-3 proteins determined by western blot

MCF-7 xenografts were established to evaluate the effect of Tan IIA on tumor growth in vivo. Tan IIA treatment (30 mg/kg, 5 times/week, 2 weeks) significantly reduced the size and weight of the tumor, in comparison to that of the vehicle control (Figs. 3a, b). Meanwhile, no significant change in body weight or pronounced side effects was observed in Tan IIA-treated mice (Fig. 3c). Western blot analysis of excised tumor tissues for PARP and caspase 3 proteins confirmed that Tan IIA treatment enhanced PARP and caspase-3 cleavage in vivo (Fig. 3d). These results indicate that Tan IIA suppressed tumor cell growth in vitro and tumor formation in vivo.

### Effects of Tan IIA on Hsp90

**Fig. 4:**
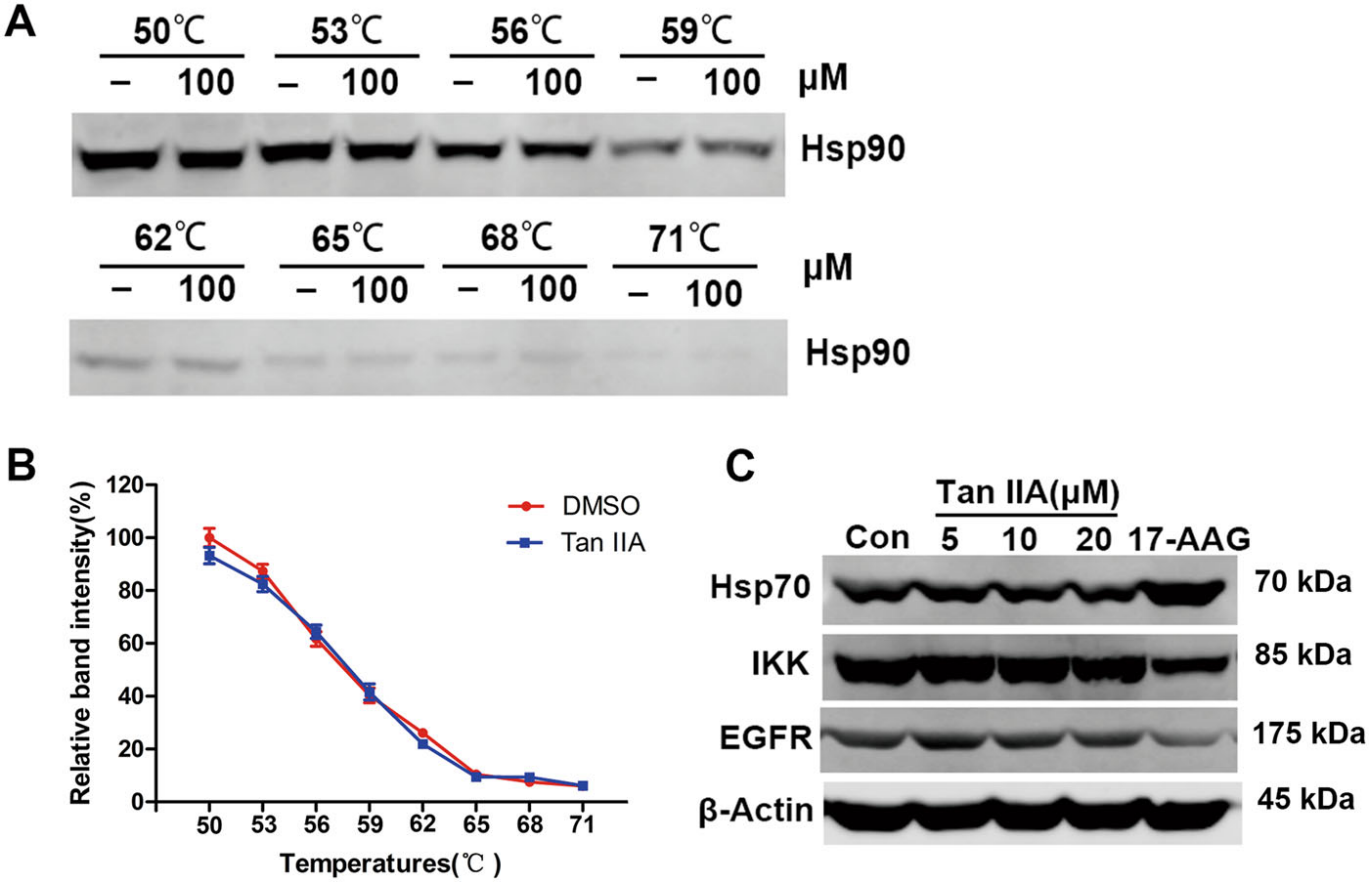
Tan IIA did not inhibit Hsp90. **a** The binding between Tan IIA (100 μM) and Hsp90 protein was examined by the thermal stabilization-based CETSA method at a range of temperatures. **b** The intensities of bands shown in (**a**) were quantified and the data are presented as mean ± S.D. of three independent experiments. **c** Western blot analysis of Hsp70 protein and Hsp90 client proteins IKK and EGFR after 24 h Tan IIA treatment. The Hsp90 inhibitor 17-AAG (10 μM) was included as a positive control

The CMAP results suggested that Tan IIA may act in a manner similar to that of Hsp90 inhibitors. The Hsp90 molecular chaperone plays an important role in tumorigenesis by regulating the maturation, stability, and activation of numerous client proteins^22^. The interaction between Tan IIA and Hsp90 protein was examined by cellular thermal shift assay (CETSA), a newly developed method that is based on the thermal stabilization of ligand-bound proteins. Heating denatures and precipitates proteins, reflected by the disappearance of soluble Hsp90 protein from lysate samples at elevated temperatures (Figs. 4a, b). The observation that there is no significant difference between solvent (DMSO) and Tan IIA treated samples suggested that Tan IIA does not bind Hsp90 protein. In general, Hsp90 inhibitors increase the expression of Hsp70 protein while decreasing the levels of Hsp90 client proteins [e.g., IκB kinase (IKK), epidermal growth factor receptor (EGFR), etc.]^23^. In MCF-7 cells, Tan IIA treatment did not affect the levels of Hsp70, IKK, or EGFR proteins (Fig. 4c). The Hsp90 inhibitor tanespimycin (17-AAG) was included as a positive control. Even though CMAP indicated that Tan IIA acted similar to 17-AAG based on gene expression profiles, Tan IIA did not induce the expression of HSPs or reduce the levels of receptor tyrosine kinases like a true Hsp90 inhibitor. These results indicate that Tan IIA does not function as an Hsp90 inhibitor.

### Tan IIA acted synergistically with the Hsp90 inhibitors 17-AAG and ganetespib

**Fig. 5:**
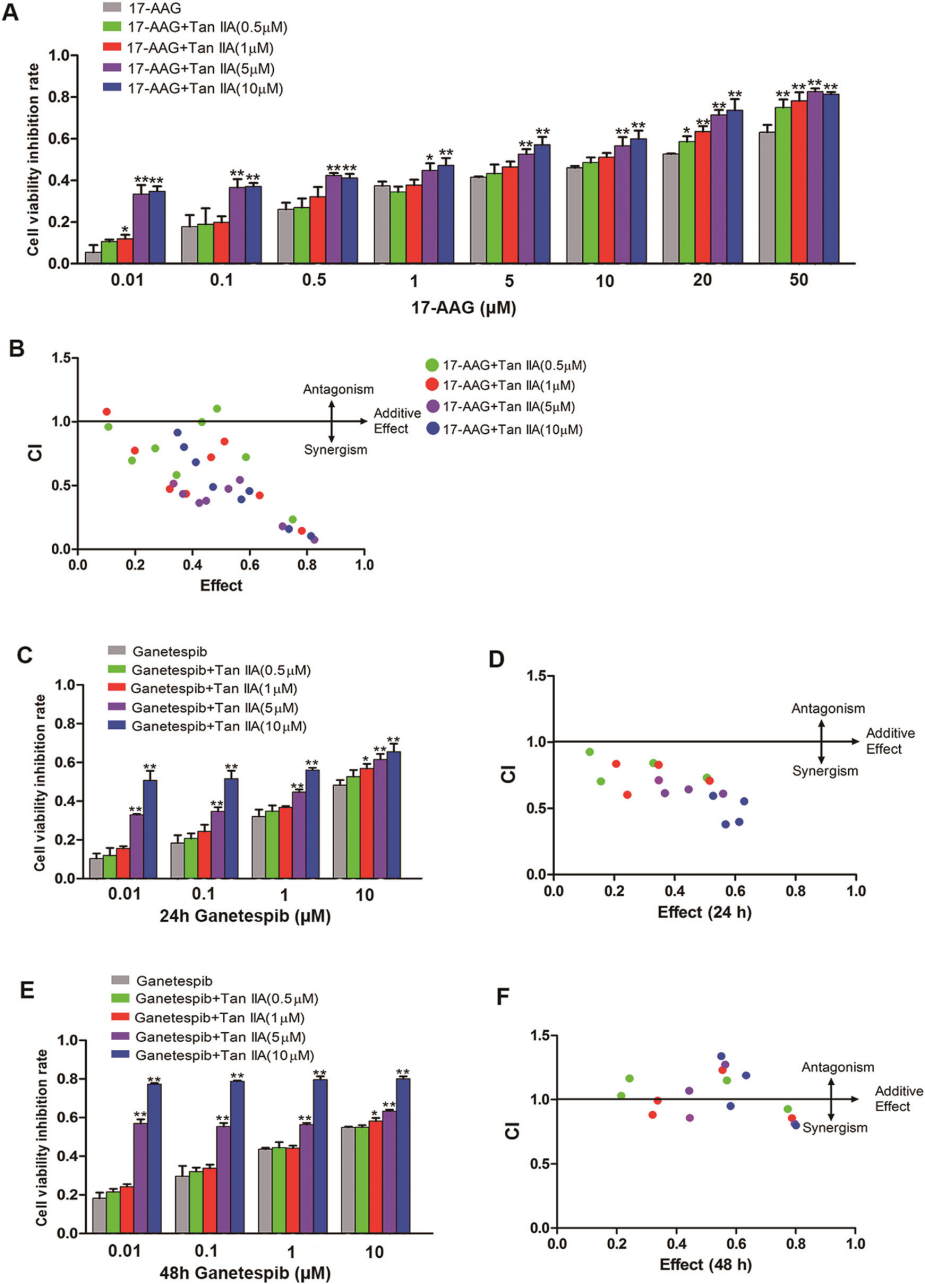
Tan IIA synergistically enhanced the anti-proliferative activity of 17-AAG and ganetespib. **a** MCF-7 cells were exposed to 17-AAG at the specified concentrations for 24 h in the presence or absence of Tan IIA (0.5, 1, 5, and 10 μM, respectively). Cell viability was determined by the CCK-8 method. **b** The CI (combination index) values for Tan IIA and 17-AAG combinations. **c** MCF-7 cells were exposed to ganetespib at the specified concentrations for 24 h in the presence or absence of Tan IIA (0.5, 1, 5, and 10 μM, respectively). **d** The CI values for Tan IIA and ganetespib combinations by treating cells for 24 h. **e** MCF-7 cells were exposed to ganetespib at the specified concentrations for 48 h in the presence or absence of Tan IIA (0.5, 1, 5, and 10 μM, respectively). **f** The CI values for Tan IIA and ganetespib combinations by treating cells for 48 h. All the data presented as inhibition rate of the untreated control (mean ± S.D. of three independent experiments). *p < 0.05 and **p < 0.01 when compared to the corresponding 17-AAG or ganetespib treatment in the absence of Tan IIA

Even though the CMAP analysis indicated that 17-AAG and Tan IIA exert similar effects on gene expression, a direct interaction between Tan IIA and Hsp90 protein was not detected. Earlier studies revealed that Hsp90 inhibitors exhibited only limited antitumor activity as a single agent, but significantly enhanced activity when combined with other antitumor agents^24^. MCF-7 cells were exposed to 17-AAG for 24 h at a range of concentrations in the presence or absence of Tan IIA at specified concentrations (0.5, 1, 5, and 10 μM, respectively) and cell viability was determined by the CCK-8 assay. When 17-AAG was in low concentrations, only 5 and 10 μM Tan IIA could significantly increase the anti-proliferative/cytotoxic activity (Fig. 5a). At higher concentrations of 17-AAG (i.e., 20 and 50 μM), Tan IIA enhanced the anti-proliferative activity of 17-AAG at all concentrations tested (Fig. 5a). The data were further analyzed to calculate a “Combination Index” (CI) using the medium-effect equation^25^. The CI values of <1, =1, and >1 are indicative of synergism, additive effects, and antagonism, respectively. As shown in Fig. 5b, most of the CI values are <1, suggesting that the combination of Tan IIA and 17-AAG produced synergistic antitumor effects in MCF-7 cells (detailed CI values in Supplementary Data 2). Lower CI values were obtained when Tan IIA was combined with higher concentrations of 17-AAG, indicating exhibited stronger synergistic effects.

17-AAG is an early generation Hsp90 inhibitor. We also assayed synergistic antitumor effects of the novel Hsp90 inhibitor ganetespib with Tan IIA. When MCF-7 cells were treated with ganetespib and Tan IIA for 24 and 48 h, Tan IIA (5 and 10 μM) could significantly increase the anti-proliferative activity of ganetespib (Fig. 5c, e). the CI values of ganetespib combined with Tan IIA are <1 at 24 h and close to 1 at 48 h (detailed CI values in Supplementary Data 3 and 4) (Fig. 5d,f). The analyzed results suggested that Tan IIA combined with ganetespib treating cells for 24 h exerted better synergistic effects than 48 h. Collectively, these observations support the potential therapeutic application of Tan IIA in combination with Hsp90 inhibitors.

### Tan IIA inhibits PKC and Ras/MAPK signaling pathways

**Fig. 6:**
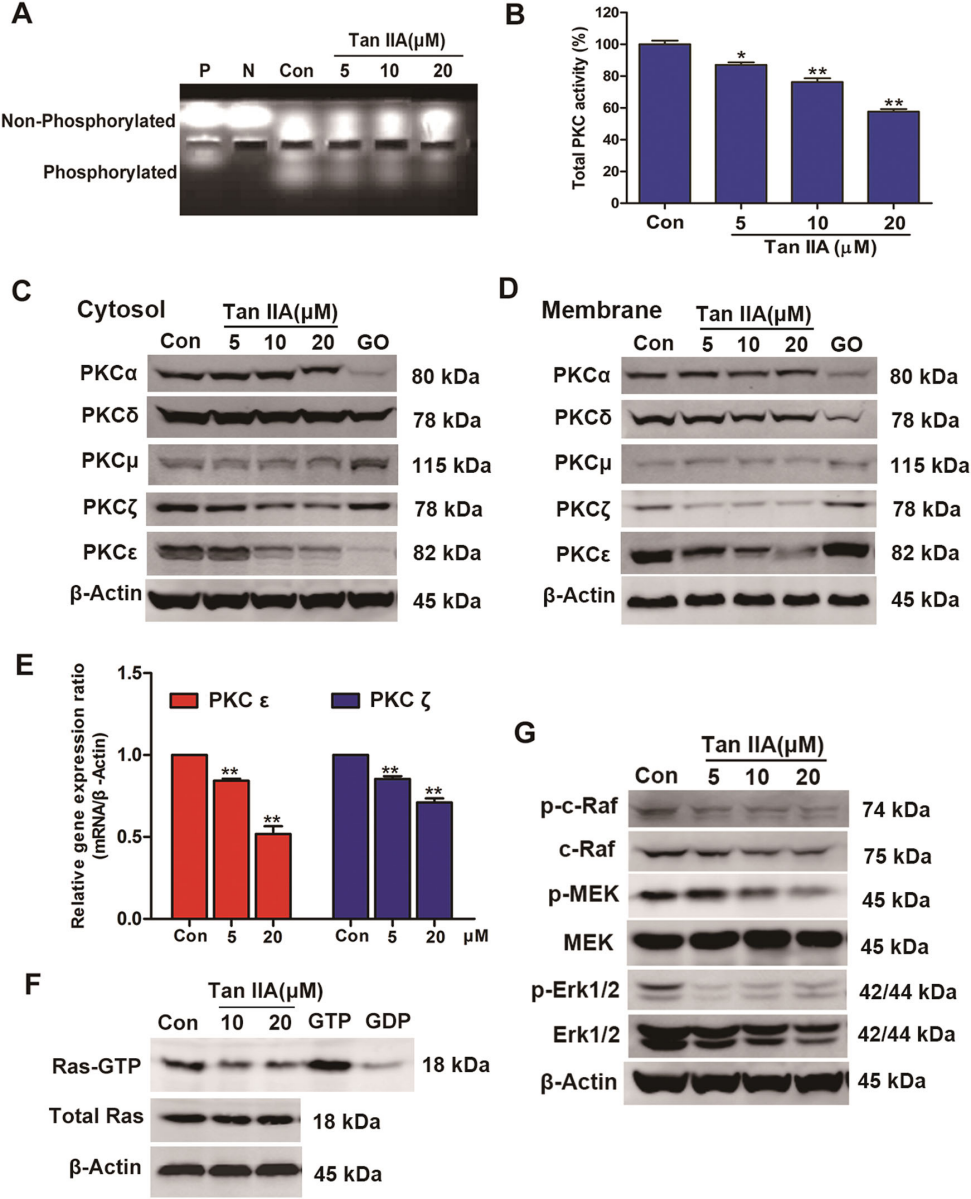
Inhibitory effects exerted by Tan IIA on PKC and Ras/MAPK pathway. **a** Lysate samples prepared from Tan IIA (5, 10, and 20 μM, respectively) treated MCF-7 cells were examined for PKC activity using the PepTag® assay. “Con” represents lysate sample from untreated MCF-7 cells, “P” for positive control (20 ng purified PKC), and “N” for negative control (water only). **b** Quantification results of the phosphorylated PepTag peptides for PKC activity. Data shown are mean ± standard deviation of three independent experiments. *p < 0.05 and **p < 0.01 when compared to the control. **c** Western blot analysis of MCF-7 cells treated with Tan IIA at the specified concentrations for the expression of cytosolic PKC isoforms. The compound Go 6983 (GO, 20 μM) was included as a positive control. **d** Similar analysis as that described in (C) except membrane fractions were used to monitor the levels of membrane PKC isoforms. **e** The expression levels of PKCε and PKCζ genes in MCF-7 cells treated with Tan IIA were determined by real-time PCR. Data shown are mean ± standard deviation of three independent experiments. *p < 0.05 and **p < 0.01 when compared to the control. **f** Ras activation monitored using a pull-down assay followed by western blot for the levels of GTP-bound active Ras in control and Tan IIA (10 and 20 μM, 24 h)-treated MCF-7 cells. The levels of total Ras and β-actin proteins were determined to serve as a reference for comparison. Lysate samples prepared from untreated control were incubated with GTP and GDP as positive and negative controls, respectively. **g** Effects of Tan IIA on the activity of the Ras/MAPK pathway. Following Tan IIA treatment of MCF-7 cells, the levels of active c-Raf (p-c-Raf), MEK (p-MEK), and Erk1/2 (p-Erk1/2) were determined by western blot. The levels of total c-Raf, MEK, and Erk1/2 proteins were also determined for comparison

The CMAP analysis revealed that Tan IIA may function as a protein kinase C (PKC) inhibitor. As a major regulator of cellular functions, PKC dysregulation has been implicated in the etiology of multiple different types of cancer^26–29^. To determine if Tan IIA inhibits PKC, a rapid non-radioactive method was used to monitor PKC activity. As indicated by the observed decrease in the levels of phosphorylated PKC substrate, Tan IIA inhibited PKC activity in a concentration-dependent manner (42% at 20 μM, Fig. 6a,b).

Protein kinase C isozymes are classified into three groups: conventional or classical PKCs (cPKCs: *α*, βI, βII, *γ*), novel PKCs (nPKCs: *δ*, *ε*, *η*, *θ*, *μ*), and atypical PKCs (aPKCs: *ζ*, λ/ι)^30^. A number of PKC isoforms are overexpressed in distinctly different forms of cancer^26^. To determine if Tan IIA can inhibit PKC expression, the levels of five representative PKC isoforms (cPKC: *α*; nPKC: *δ*, *ε*, *μ*; and aPKC: *ζ*) were determined by western blot in treated MCF-7 cells. Tan IIA treatment (24 h) significantly reduced the levels of PKCζ and PKCε in both the cytosolic and membrane fractions, without affecting the levels of PKC isoforms *α*, *δ* or *μ* (Figs. 6c, d). These observations suggest that Tan IIA selectively inhibits the expression of PKCζ and PKCε. Furthermore, real-time PCR determined that the expression levels of PKCε and PKCζ genes in MCF-7 cells also significantly decreased by Tan IIA (Fig. 6e).

Ras proteins are key regulators of cell growth, differentiation, and survival, and oncogenic activation of Ras is associated with the etiology and progression of cancer^31^. Emerging evidence indicates that PKC suppression sensitizes tumor cells with oncogenically activated Ras to apoptosis^18,19,32^. Mitogen-activated protein kinase (MAPK) acts downstream of Ras in the Ras/MAPK pathway that plays an important role in tumorigenesis and malignant progression^33,34^. To determine the effects of Tan IIA on the downstream targets regulated by PKC, we examined the activation status of Ras and major kinases from the Ras/MAPK pathway (c-Raf, MEK, and Erk1/2). As anticipated, Tan IIA treatment significantly reduced the amount of active GTP-bound Ras, without affecting total Ras protein levels (Fig. 6f). Further, Tan IIA inhibited the Ras/MAPK pathway, reflected by concentration-dependent decreases in the levels of phosphorylated c-Raf, MEK and Erk1/2, respectively (Fig. 6g). These results support the notion that Tan IIA exerts an inhibition on the Ras/MAPK signaling pathway.

### Tan IIA inhibits PI3K/Akt/mTOR signaling and induces cell cycle arrest and autophagy

**Fig. 7:**
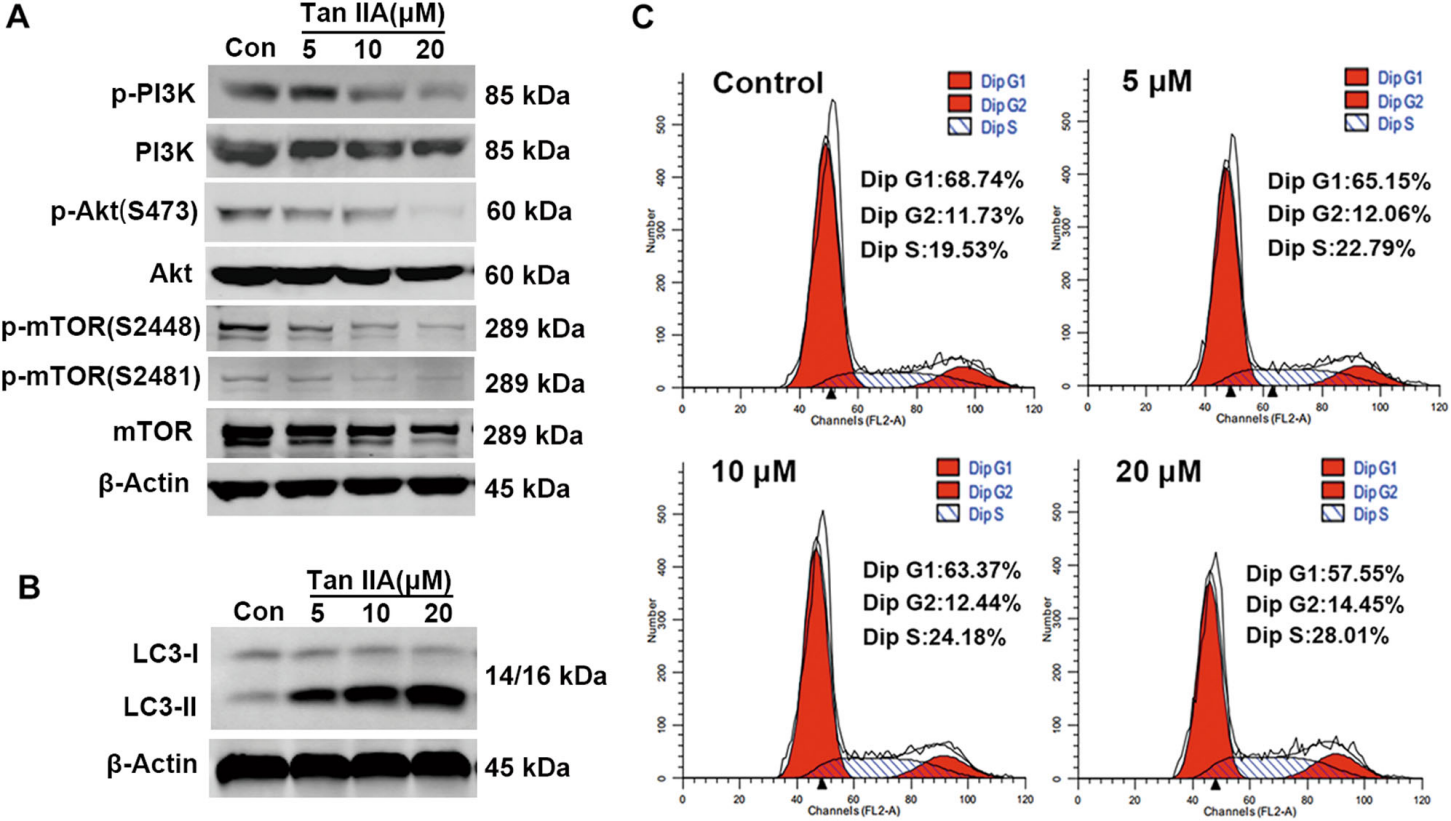
Tan IIA inhibited the PI3K/Akt/mTOR pathway and induced cell cycle arrest and autophagy in MCF7 cells. **a** The activity of the PI3K/Akt/mTOR pathway in Tan IIA treated MCF-7 cells was monitored by measuring the levels of active PI3K (p-PI3K), Akt (p-Akt), and mTOR (p-mTOR), and the levels of total proteins for each kinase. **b** Flow cytometry-based analysis of cell cycle distribution in control and Tan IIA treated MCF-7 cells. **c** To determine the extent of autophagy, western blot analysis was performed to detect LC3-II proteins in lysate samples prepared from control and Tan IIA-treated MCF-7 cells. For comparison, western blot results of LC3-I and β-actin were also included

Other potential Tan IIA targets identified in the CMAP analysis include PI3K and mTOR. As an essential serine/threonine kinase, mTOR belongs to the PI3K-related kinase family^35,36^. Dysregulated PI3K/Akt/mTOR signaling is involved in tumor cell growth, proliferation, apoptosis, survival, invasion, and metastasis^37–39^. The effects of Tan IIA on the PI3K/Akt/mTOR pathway were examined by western blot and the results are shown in Fig. 7a. In MCF-7 cells, Tan IIA treatment decreased the levels of activated PI3K (p-PI3K), Akt (p-Akt), and mTOR (p-mTOR) without inducing pronounced changes in the total protein levels of each kinase (Fig. 7a). These observations suggest that Tan IIA inhibits the activation of the PI3K/Akt/mTOR pathway, not the expression of enzymes that are components of this signaling pathway. At the cellular level, the PI3K/Akt/mTOR pathway regulates cell cycle progression, autophagy, and programmed cell death. The effects of Tan IIA on cell cycle progression and autophagy were examined in MCF-7 cells. Flow cytometry-based DNA content analysis revealed that Tan IIA induced cell cycle arrest at the S and G2 phase, indicated by the increase in the percentage of cells in S and G2 phase and the decrease in the percentage of cells in G1 phase (Fig. 7b). To assess the effect of Tan IIA on autophagy, the expression of LC3 (microtubule-associated protein 1A/1B-light chain 3) was monitored by western blot. During autophagy, LC3-I (autophagy-inactive, cytosolic) is converted into LC3-II (autophagy-active, membrane bound, LC3-phosphatidylethanolamine conjugate) and the appearance of LC3-II serves as a marker for autophagy^40^. Exposure to Tan IIA induced a marked concentration-dependent increase of LC3-II protein in MCF-7 cells (Fig. 7c). These results indicate that Tan IIA induces cell cycle arrest and autophagy. Both processes are under the tight regulation of the PI3K/Akt/mTOR pathway.

## Discussion

Many pharmacologically active compounds hit more than one molecular target^41^. Previous studies suggest that the antitumor effects of Tan IIA may result from the perturbation of multiple potential targets/pathways, yet its underlying molecular mechanisms remain unresolved. Gene expression profile-based CMAP database analysis has been used to elucidate the molecular mechanisms of new therapeutic candidates and to discover new drugs. For example, the application of CMAP database analysis led to the discovery of a new function of celastrol, which may yield potential weight loss product^42^. Rather than focusing on one target gene, chemogenomics-based approaches can establish a suite of drug candidate gene expression signatures that can be used to query the CMAP database for similarities with agents that function through known targets/mechanisms. Our initial CMAP analysis identified positive correlations in gene expression signatures between Tan IIA and Hsp90 inhibitors (e.g., tanespimycin and geldanamycin), PKC inhibitor (rottlerin), PI3K inhibitor (LY-294002), and mTOR inhibitor (sirolimus). The targets/pathways regulated by these active hits all play important roles in tumorigenesis and malignant progression. Further investigation revealed that Tan IIA stalled tumor growth by inducing cell cycle arrest and autophagy, through the inhibition of PKC, Ras/MAPK, and PI3K/Akt/mTOR pathways.

Protein kinase C-regulated signaling pathways control cellular functions that range from gene expression, cell-cycle, proliferation, differentiation, apoptosis, and survival, to tumor cell migration^26^. The overexpression of PKC isoforms has been observed in breast cancer cell lines as well as in breast tumors, which may serve as both prognostic biomarkers and indicators of treatment responsiveness^43^. Patients with PKCα-positive breast tumors exhibited increased mortality relative to those that are PKCα-negative^44^, and breast cancer cell lines with high levels of PKCα (e.g., MDA-MB-231) proliferate faster than those with low PKCα levels (e.g., T47D)^45^. Increased PKCε expression correlates with advanced disease stage, poor disease-free progression and overall survival in breast cancer patients, and the downregulation of PKCε reduced tumor growth and stalled metastatic progression in MDA-MB-231 cell-based models^46^. Overexpression of PKCζ induced phenotypic alterations in immortalized mammary epithelial cells that are associated with malignant transformation and tumor progression^43^. Further, PKCμ has been implicated in tumor cell invasion and PKCδ upregulation contributes to antiestrogen resistance^47,48^. This study revealed a novel mechanism for the antitumor activity of Tan IIA through the inhibition of PKC signaling. Not only did Tan IIA inhibit the enzymatic activity of PKC, it also selectively suppressed the expression of PKCζ and PKCε isoforms. The inhibition of PKC by Tan IIA is transmitted to the overall dampening of the Ras/MAPK pathway that regulates cell proliferation and growth. These observations clearly delineate the contribution of the PKC/Ras/MAPK signaling to the antitumor action of Tan IIA.

While the CMAP analysis identified positive connections between Tan IIA and Hsp90 inhibitors (tanespimycin and geldanamycin), Tan IIA did not bind Hsp90 protein. More than 200 proteins are Hsp90 client proteins, specifically, Hsp90 regulates the stabilization and activation of these proteins. Tan IIA inhibited a group of Hsp90 client proteins that include Akt, c-Raf, and MEK, but not others such as IKK and EGFR. This type of “overlap” in targets/pathways may result in the positive connection between Tan IIA and tanespimycin (17-AAG) identified by the CMAP analysis. An interesting finding of further study showed that Tan IIA and 17-AAG had synergistic antitumor efficacy on MCF7 cells, especially high concentrations of 17-AAG could exhibit stronger synergistic effects with Tan IIA. These results suggested that Tan IIA is potential to increase the expected maximal antitumor activity of 17-AAG alone, which is of clinic interest. Moreover, Tan IIA also enhanced the novel Hsp90 inhibitor ganetespib antitumor activity and produced better synergistic effects at 24 h. 17-AAG was once conducted clinical evaluation for breast cancer treatment^49^, and the novel Hsp90 inhibitor ganetespib is undergoing clinical trials for breast cancer treatment^50^. Our findings suggest that Tan IIA may enhance the efficacy of Hsp90 inhibitors 17-AAG and ganetespib, which support further exploration of a combination therapy for breast cancer treatment.

Differences between cell lines, experimental conditions, model systems, and data analyses may introduce inconsistency in the characterization and mechanistic investigation of bioactive molecules. While the integrated gene expression profile and CMAP database-based bioinformatic mining shed light on candidate targets/pathways for the potential anticancer activity of Tan IIA, it is essential to verify these predictions in experimental model systems. For example, we confirmed the inhibition of PI3K/Akt/mTOR signaling by Tan IIA in MCF-7 breast tumor cells. This corroborates other studies that suggest Tan IIA may inhibit PI3K/Akt/mTOR signaling in gastric carcinoma and prostate cancer models^51,52^, and serves as a validation for the efficacy and accuracy of our chemogenomics-based strategy. This gene expression profile-based bioinformatic approach can generate new hypotheses that can be tested in experimental systems to resolve the molecular mechanisms of drugs and other pharmacologically active agents.

## Materials and methods

### Chemicals and antibodies

Tan IIA was purchased from the National Institute for the Control of Pharmaceutical and Biological Products (Beijing, China), Go 6983, tanespimycin (17-AAG) and ganetespib from Medchem Express (Monmouth Junction, NJ, USA). Antibodies specific for β-actin (#4970), Hsp70 (#4872), IKKα (#2682), EGFR (#4267), PKCα (#2056), PKCδ (#9616), PKCμ (#2052), PKCζ (#9368), PKCε (#2683), Phospho-c-Raf (#9427), c-Raf (#9422), Phospho-MEK1/2 (#9154), MEK1/2 (#8727), Phospho-Erk1/2 (#4370), Erk1/2 (#9102), Phospho-PI3K (#4228), PI3K (#4257), Phospho-Akt (#4060), Akt (#9272), Phospho-mTOR (S2448) (#5536), Phospho-mTOR (S2481) (#2974), mTOR (#2983), LC3B (#3868), Bcl-2 (#2872), PARP (#9542) and cleaved caspase-3 (#9661) were obtained from Cell Signaling Technology (Danvers, MA, USA). Anti-Hsp90 (#ab13492) and anti-Ras (#ab52939) antibodies were from Abcam (Cambridge, UK).

### Cell culture

Human breast cancer MCF-7 cells (Product No. HTB-22, ATCC, Manassas, VA, USA) were maintained in Minimum Essential Medium/Earle’s Balanced Salt Solution (MEM/EBSS) (Hyclone, Logan, UT, USA) supplemented with 10% fetal bovine serum (Biological Industries, Cromwell, CT, USA), 1 mM sodium pyruvate (Sigma, St. Louis, MO, USA), 0.1 mM MEM non-essential amino acids (Sigma, St. Louis, MO, USA), 100 unit/mL penicillin and 100 mg/mL streptomycin (Gibco, Carlsbad, CA, USA) at 37 °C in a humidified environment that contains 5% CO_2_: 95% Air.

### Cell viability assay

Cell viability was determined using a commercial kit [Cell Counting Kit-8 (CCK-8), Dojindo, Kumamoto, Japan], following manufacturer’s instructions. Cells plated in 96-well plates were treated with Tan IIA at the specified concentrations for 12, 24, and 48 h, respectively. The incubation continued for another 3 h after the addition of CCK-8 and the absorbance at 450 nm measured using a microplate reader (BioTek Instruments, Winooski, VT, USA). For combination studies, cells were treated with different combinations of Tan IIA and 17-AAG at specified concentrations for 24 h. MCF-7 cells were also treated with different combinations of Tan IIA and ganetespib at specified concentrations for 24 and 48 h. The CI was calculated using a modified combination index curve based on the medium-effect equation^25^.

$$CI = \frac{{(D)_1}}{{(D_\chi )_1}} + \frac{{(D)_2}}{{(D_\chi )_2}}$$

(D_*x*_)_1_ is for D_1_ “alone” that inhibits a system by *x*%, and (D_*x*_)_2_ is for D_2_ “alone” that inhibits a system by *x*%.

### Cell morphological analysis

MCF-7 cells plated in 6-well plates were exposed to Tan IIA for 24 h, and observed for morphological changes and characteristics indicative of either apoptosis or necrosis under a phase-contrast microscope (Leica Microsystems, Inc., Germany). Images were captured at 100× magnification.

### Analyses of apoptosis and cell cycle by flow cytometry

MCF-7 cells plated in 6-well plates were exposed to Tan IIA for 24 h at 5, 10, and 20 μM, respectively. Both floating and adherent cells were harvested for further analysis. The Annexin V-FITC/PI apoptosis detection kit (BD Biosciences, San Jose, CA, USA) was used to detect apoptosis and the cell cycle and apoptosis analysis kit (Beyotime, Haimen, China) for cell cycle analysis, following the manufacturer’s instructions.

### Cellular thermal shift assay (CETSA)

Cellular thermal shift assay was performed as described^53,54^. Briefly, MCF-7 cells were harvested, washed with PBS three times, and lysed in PBS supplemented with protease inhibitors following three cycles of freeze-thaw. Cell lysate samples were subjected to centrifugation, the supernatant collected, and aliquots were treated with Tan IIA (100 μM) or solvent control (DMSO) for 20 min at room temperature. Samples from either compound or solvent treated cells were divided into aliquots in a volume of 50 μL and heated at a range of temperatures (50, 53, 56, 59, 62, 65, 68, and 71 °C, respectively) for 3 min, cooled for 3 min at room temperature, and then kept on ice. The heated samples were subjected to centrifugation and the supernatant (soluble fractions) analyzed by western blot.

### PKC activity assay

The PepTag^®^ assay for non-radioactive detection of protein kinase C (Promega, Madison, WI, USA) was used to monitor PKC activity, following the manufacturer’s instruction. The phosphorylated and non-phosphorylated forms of the PepTag^®^ peptides were separated by agarose gel electrophoresis, photographed using a G:BOX gel imaging system (Gene Co., Hong Kong, China), and the negatively charged phosphorylated bands were excised for quantification by measuring absorbance at 570 nm using a microplate reader (BioTek Instruments).

### Ras activity assay

Ras activation was monitored by measuring the levels of active GTP-bound state Ras, using the Ras pull-down activation assay biochem kit (Cytoskeleton, Denver, CO, USA). Briefly, cell lysate samples (200 μg each) were incubated with Raf1-Ras-binding domain bound agarose beads. The active GTP-bound Ras protein was eluted into Laemmli sample buffer and analyzed by western blot. The controls for affinity purification were MCF-7 cell lysates mixed with GDP (negative) or GTPγS (positive). Western blot was used to detect both total and active Ras proteins.

### RNA extraction and real-time PCR analysis

MCF-7 cells exposed to Tan IIA (5 and 20 μM) for 24 h and total mRNA extracted by using the Trizol reagent (Invitrogen, CA, USA) according to the manufacturer’s protocol. Total mRNA was reverse transcribed into cDNA using PrimeScript RT reagent Kit (Takara, Shiga, Japan) following the manufacturer’s protocol. The quantitative real-time PCR analysis was performed on QuantStudio 6 Flex real-time PCR system (Applied Biosystems, CA, USA) using SYBR Premix Ex Taq II (Takara, Shiga, Japan). β-Actin was used as the reference gene. The forward and reverse primers used were listed in Supplementary Table 1.

### Western blot

Total cellular proteins were extracted with radioimmunoprecipitation assay buffer, membrane and cytosolic proteins extracted with a membrane and cytosol protein extraction kit (Beyotime, Haimen, China). Protein concentrations were determined using a BCA protein assay kit (Beyotime, Haimen, China). Standard procedures were applied for western blot analysis. After blocking with 5% nonfat dry milk, the membranes were incubated with primary antibodies at manufacturers’ recommended dilutions at 4 °C overnight, and the incubation continued for another 60 min at room temperature after the addition of a goat anti-rabbit or donkey anti-mouse secondary antibody (IRDye 800, LI-COR, Lincoln, NE, USA). The immunoreactive bands were scanned using an Odyssey Infrared Imaging System (LI-COR).

### Xenograft models

All animal studies were performed according to the institutional ethical guidelines of animal care and were approved by the Committee on the Ethics of Animal Experiments of the Second Military Medical University, China. Female BALB/c-nu nude mice (4-week-old) were obtained from the Shanghai SLAC Laboratory Animal Co., Ltd. (Shanghai, China). The flank xenografts were established by subcutaneous injections of 8 × 10^6^ cells. Following a two week inoculation period, the tumor-bearing mice were randomly divided into Tan IIA treatment and control groups. The treatment group was injected with 30 mg/kg Tan IIA five times/week for 2 weeks and the control group was injected with the same volume of saline. Body weights and tumor size were recorded two times per week.

### Statistics analysis

Statistical analysis was performed with one-way ANOVA followed by Tukey’s post hoc test when comparing multiple independent groups, and unpaired *t*-test when comparing two different groups. Values of *p* < 0.05 were considered statistically significant. All statistical analyses were performed using SPSS software (version 16.0, SPSS, Chicago, IL, USA).

## Electronic supplementary material

Figure 1S

Supplementary Data 1

Supplementary Data 2

Supplementary Data 3

Supplementary Data 4

Supplementary Data 5

Supplementary Table 1

Supplementary Figure Legend
