# Supplementary figures and images for: The antitumor natural product tanshinone IIA inhibits protein kinase C and acts synergistically with 17-AAG
Source: Cell Death Dis. 2018 Feb 7;9(2):165. doi: 10.1038/s41419-017-0247-5 (PMC5833361; doi:10.1038/s41419-017-0247-5)

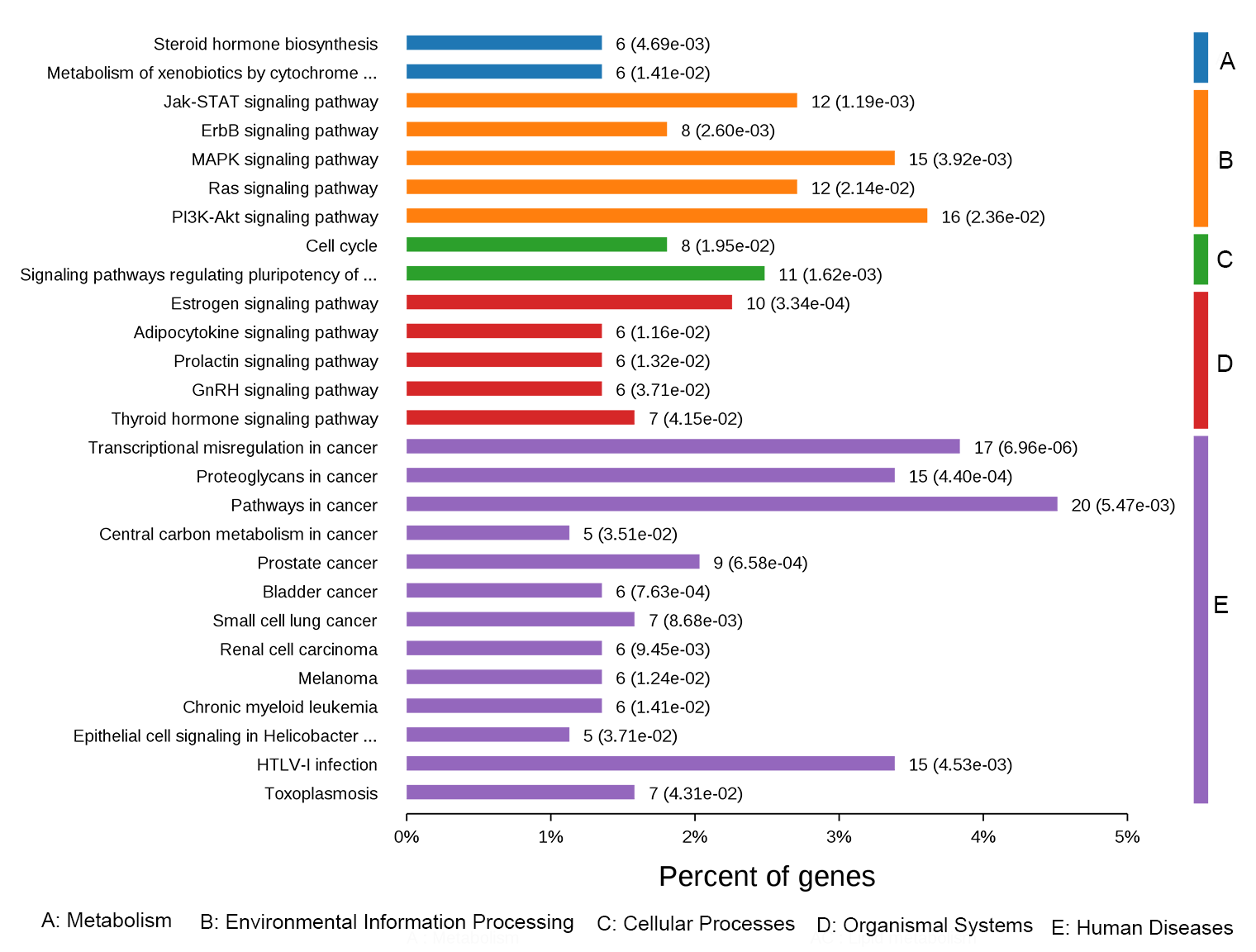

Supplement: Supplementary file 1 — Figure 1S [file 41419_2017_247_MOESM1_ESM.tif]
