# Supplementary material for: The antitumor natural product tanshinone IIA inhibits protein kinase C and acts synergistically with 17-AAG
Source: Cell Death Dis. 2018 Feb 7;9(2):165. doi: 10.1038/s41419-017-0247-5 (PMC5833361; doi:10.1038/s41419-017-0247-5)
Supplement: Supplementary file 7 — Supplementary Table 1 [file 41419_2017_247_MOESM7_ESM.docx]

**Supplementary Table 1.**

Primers sequences for qPCR analysis

| Gene | Forward primer | Reverse primer |
| --- | --- | --- |
| PKC ε | TCGGGTGAAGCCCCTAAAGA | GGCTGCCGAAGATAGGTGG |
| PKC ζ | ATGACGAGGATATTGACTGGGT | CAGGAGTGTAATCCGACCAGG |
| β-Actin | TGACGTGGACATCCGCAAAG | CTGGAAGGTGGACAGCGAGG |
