# Supplementary material for: The antitumor natural product tanshinone IIA inhibits protein kinase C and acts synergistically with 17-AAG
Source: Cell Death Dis. 2018 Feb 7;9(2):165. doi: 10.1038/s41419-017-0247-5 (PMC5833361; doi:10.1038/s41419-017-0247-5)
Supplement: Supplementary file 8 — Supplementary Figure Legend [file 41419_2017_247_MOESM8_ESM.docx]

**Supplementary Figure Legend**

**Fig. S1 Distribution of enriched KEGG pathway.** Columns refer to related pathway.
